# *Pax3* lineage-specific deletion of *Gpr161* is associated with spinal neural tube and craniofacial malformations during embryonic development

**DOI:** 10.1242/dmm.050277

**Published:** 2023-11-28

**Authors:** Sung-Eun Kim, Pooja J. Chothani, Rehana Shaik, Westley Pollard, Richard H. Finnell

**Affiliations:** ^1^ Department of Pediatrics, Dell Pediatric Research Institute, Dell Medical School, University of Texas at Austin, Austin, TX 78723, USA; ^2^ Center for Precision Environmental Health, Department of Molecular and Cellular Biology, Baylor College of Medicine, Houston, TX 77030, USA; ^3^ Departments of Molecular and Human Genetics and Medicine, Baylor College of Medicine, Houston, TX 77030, USA

**Keywords:** Gpr161, *Pax3*, Wnt/β-catenin signaling, Craniofacial development, Spinal neural tube

## Abstract

Sonic hedgehog (Shh) signaling is the morphogen signaling that regulates embryonic craniofacial and neural tube development. G protein-coupled receptor 161 (Gpr161) is a negative regulator of Shh signaling, and its inactivation in mice results in embryo lethality associated with craniofacial defects and neural tube defects. However, the structural defects of later embryonic stages and cell lineages underlying abnormalities have not been well characterized due to the limited lifespan of *Gpr161* null mice. We found that embryos with *Pax3* lineage-specific deletion of *Gpr161* presented with tectal hypertrophy (anterior dorsal neuroepithelium), cranial vault and facial bone hypoplasia (cranial neural crest), vertebral abnormalities (somite) and the closed form of spina bifida (posterior dorsal neuroepithelium). In particular, the closed form of spina bifida was partly due to reduced *Pax3* and *Cdx4* gene expression in the posterior dorsal neural tubes of *Gpr161* mutant embryos with decreased Wnt signaling, whereas Shh signaling was increased. We describe a previously unreported role for Gpr161 in the development of posterior neural tubes and confirm its role in cranial neural crest- and somite-derived skeletogenesis and midbrain morphogenesis in mice.

## Teaser

**Summary:** Using *Gpr161* conditional knockout mice with *Pax3-Cre* as a model for closed spina bifida, this study highlights the involvement of *Gpr161*, *Pax3* and Wnt/β-catenin signaling in spinal neural tube development.

## INTRODUCTION

The embryonic neural tube is the precursor of the brain and spinal cord, and its development involves highly coordinated multiple cellular processes and signaling events (Nikolopoulou et al., 2017). The failure of neurulation results in neural tube defects (NTDs), a family of congenital malformations that encompass a wide spectrum of phenotypic malformations including anencephaly, spina bifida (SB), craniorachischisis and encephalocele (Ravi et al., 2021; Wilde et al., 2014). Although NTDs are the second most prevalent structural congenital malformation in humans, their complex etiology is not yet thoroughly understood. NTDs are not always isolated malformations, as they are, on occasion, associated with increased risks for Chiari II malformations (Williams, 2008), Joubert syndrome (Vogel et al., 2012) and Waardenburg syndrome (Hart and Miriyala, 2017), suggesting shared genetic etiology and/or pathogenesis of these syndromes with NTDs. In addition, there are several subtypes of SB, including myelomeningocele, meningocele, closed spinal dysmorphisms and SB occulta (Copp et al., 2015). Some of these subtypes are considered to be the closed form of SB due to the skin-covered dysmorphism of the spinal cord and as they result primarily from post-neurulation multicellular defects. In humans, closed SB, such as terminal myelocystocele and lipomyeolomeningocele, is often accompanied with the neurologically deficit, orthopedic symptoms with musculoskeletal issues (Copp et al., 2013). Yet, any genetic variants from human closed SB have not been well explored and few mouse models (Fernandez-Santos et al., 2023) have been reported that well represent human closed SB and to study the pathological development of closed SB. Therefore, the molecular and genetic pathogenesis of the closed forms of SB are, especially, not well understood.

Morphogen signaling, such as retinoic acid, sonic hedgehog (Shh), bone morphogenetic protein (BMP) and canonical Wnt/β-catenin signaling, plays a critical role in tissue patterning and organ morphogenesis during embryonic development (Briscoe and Small, 2015). Shh and canonical Wnt/β-catenin signaling regulate dorsoventral and anterior-posterior patterning in craniofacial development and neural tube morphogenesis (Andrews et al., 2019; Le Dreau and Marti, 2012). Specifically, the Shh morphogen is secreted from the neuroectoderm of the ventral brain, facial ectoderm and branchial endoderm and regulates the survival of cranial neural crest cells (CNCCs) during craniofacial development (Ahlgren and Bronner-Fraser, 1999; Nasrallah and Golden, 2001). Additionally, the Shh morphogen specifies ventral neural identities via being secreted from the floor plate or the notochord, whereas the Wnt morphogen determines dorsal neural identities as it is secreted from the roof plate during neural tube morphogenesis. How Shh and Wnt are affected reciprocally at the molecular level despite their conflicting roles during neural tube development has not been well explored. Therefore, any mutations in genes of the Shh and Wnt signaling pathways, including smoothened (*Smo*) (Jeong et al., 2004), suppressor of fused (*Sufu*) (De Mori et al., 2017; Lu et al., 2014; Svard et al., 2006), and protein kinase A (*Pka*, also known as *Prkaca*) (Huang et al., 2002; Zhu et al., 2005), are associated with craniofacial defects and NTDs in both humans and mice.

Pax3 is a paired box motif-containing transcription factor that is primarily expressed in the dorsal neuroepithelium of the neural tube, in pre-migratory neural crest cells and in the pre-somitic mesoderm in developing embryos (Goulding et al., 1991). Several *Pax3* mutant mice with NTDs, including *Splotch* mice with mutant alleles of *Pax3*, such as *Sp* (Epstein et al., 1993), *Sp^2H^* (Epstein et al., 1991) and *Sp^d^* (Vogan et al., 1993), have been reported. Among them, *Sp* is considered as an animal model for Waardenburg syndrome type 1 with the defective allele *Pax3^Rwa^* (Ohnishi et al., 2017), which included NTDs in the phenotype. In addition, *Pax3* mutant mice presented with cardiac outflow tract septation defects secondary to aberrant cardiac neural crest cell migration and skeletal muscle defects (Franz, 1993), supporting the role of *Pax3* in neural crest cell derivatives. In humans, genetic modification of *PAX3* is associated with the etiology of Waardenburg syndrome and SB (Hol et al., 1995; Nye et al., 1998), suggesting a pathogenic role of the *PAX3* gene in both diseases.

G protein-coupled receptor 161 (Gpr161) is a bona fide negative regulator of Shh signaling in multiple developmental and cellular contexts (Mukhopadhyay et al., 2013). It is primarily localized in the primary cilia for its suppressive role, and activates PKA by increasing cAMP levels, thereby promoting Gli3 processing for the transcriptional inactivation of Shh target genes in the absence of the Shh signal. *Gpr161* null mice are embryonic lethal by embryonic day (E) 10.5, and they present with cranial defects and posterior NTDs with full penetrance, along with facial and limb bud defects. *Gpr161* hypomorphic mutant mice displayed spinal NTDs and cataracts (Li et al., 2015; Matteson et al., 2008). Additionally, *Gpr161* conditional knockout (cKO) mice with various *Cre* lines, including *Wnt1-Cre*, *Nestin-Cre*, *GFAP-Cre* and *Prx1-Cre*, showed *Gpr161* depletion-mediated developmental defects at later embryonic stages, including intramembranous/endochondral skeletal defects (Hwang et al., 2018; Kim et al., 2021), facial defects (Hwang et al., 2021; Kim et al., 2021), forebrain/midbrain abnormalities (Kim et al., 2021; Shimada et al., 2019), ventriculomegaly (Shimada et al., 2019) and limb formation/patterning defects (Hwang et al., 2018), which primarily focus on craniofacial and limb development. *GPR161* genetic variants have been identified in patients with SB (Kim et al., 2019) and those with pituitary stalk interruption syndrome (Karaca et al., 2015). However, the role of Gpr161 in spinal neural tube development remains largely unknown, despite its genetic implication in patients with SB and in *Gpr161* null and hypomorphic SB mouse models. Primarily because the *Gpr161* null embryos were lethal by E10.5 and the penetrance of hypomorphic mice was only 50%, efforts to fully understand the development of SB by *Gpr161* mutations using existing mouse models were limited.

In this study, we investigated the role of Gpr161 in the cranial neural crest and dorsal neural progenitor lineages during mouse embryonic development, using *Gpr161* cKO mice with *Pax3-Cre*. We identified that *Gpr161^f/f^;Pax3-Cre* (hereafter *Gpr161* cKO) mice presented with craniofacial defects, cranial vault/facial and vertebral skeletal defects, and spinal neural tube malformations. In addition, we observed that *Pax3* gene expression was downregulated partly via inhibition of Wnt/β-catenin signaling in the dorsal spinal neural tubes of *Gpr161* cKO mice, which suggests a molecular mechanism for Gpr161-mediated spinal neural tube development in mice.

## RESULTS

### *Gpr161* conditional deletion in cranial neural crest and dorsal neural progenitor lineages results in craniofacial defects and spinal malformations

**Fig. 1.**
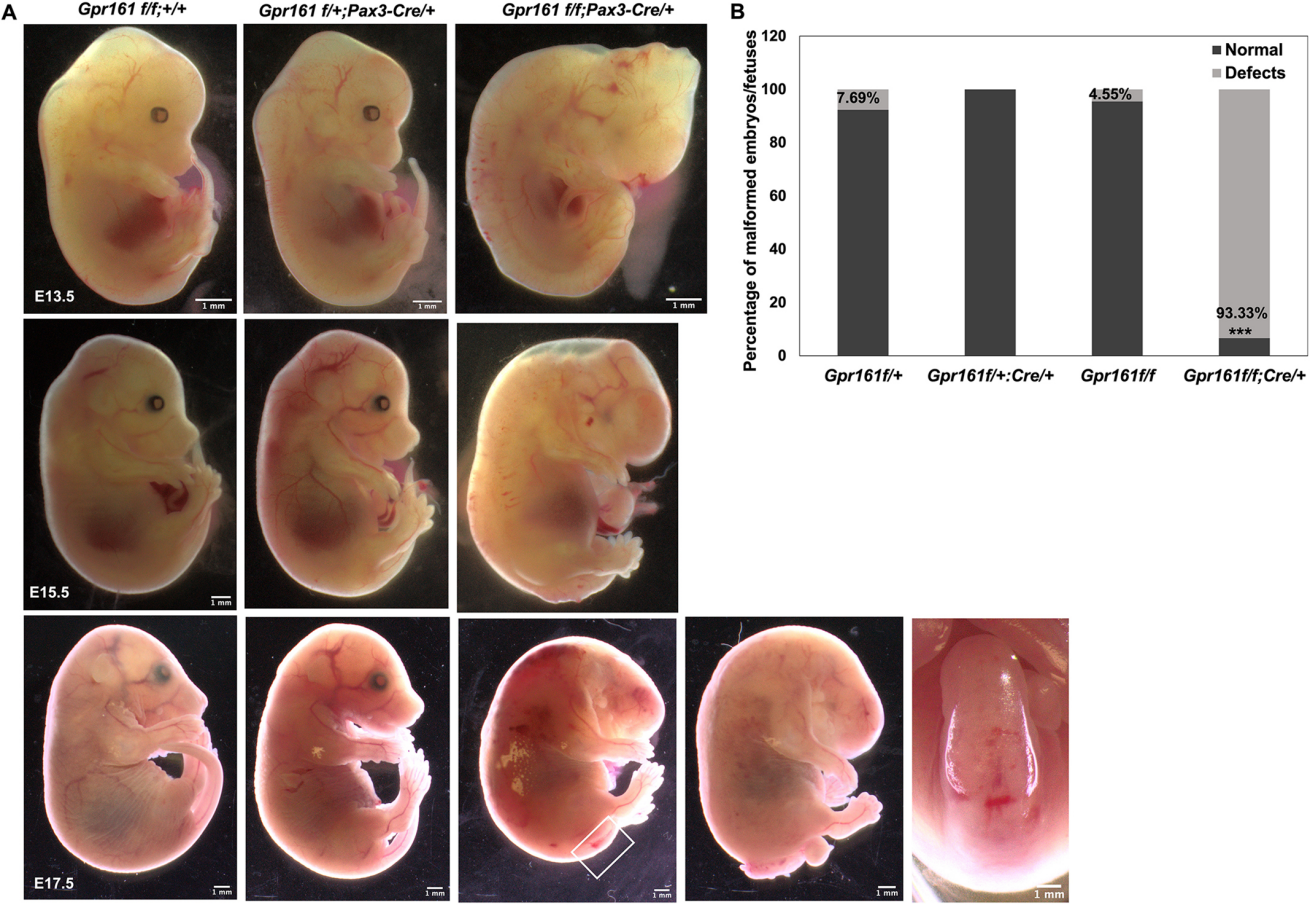
***Gpr161* depletion in *Pax3* lineages results in craniofacial defects and spinal neural tube malformation.** (A) Gross morphology of fetuses with *Gpr161^f/f^* (*flox* control), *Gpr161^f/+^;Pax3-Cre/+* (*Cre* control) and *Gpr161^f/f^;Pax3-Cre/+* (*Gpr161* cKO) at E13.5 (*n*=4), E15.5 (*n*=3) and E17.5 (*n*=8). The dorsal view of the white box in the *Gpr161* cKO embryo at E17.5 is shown in the last panel. Scale bars: 1 mm. (B) Percentage of malformed embryos/fetuses. Statistical analysis based on Table 1. Two-sample proportion test was performed between *flox* control versus *Gpr161* cKO and *Cre* control versus *Gpr161* cKO. Both comparisons showed statistically significant differences (***P<0.001, α<0.05).

Previous studies demonstrated that *Gpr161* mutant mice present with craniofacial malformations during mouse embryonic development (Hwang et al., 2021; Kim et al., 2019, 2021; Mukhopadhyay et al., 2013). In addition, *Gpr161* knockout (KO) embryos presented with delayed posterior neuropore closure (Kim et al., 2019; Mukhopadhyay et al., 2013), which potentially developed into spinal neural tube malformations at later fetal stages. As *Gpr161* KO embryos were lethal by E10.5, we used *Gpr161* cKO mice along with *Pax3-Cre* lines to define the lineage-specific role of Gpr161 during craniofacial and spinal neural tube development at later stages of fetal development. For *Pax3-Cre*, *Cre* was inserted in the *Pax3* locus (Engleka et al., 2005), and Cre was expressed specifically in the dorsal neural tube, somite and facial structure in developing mouse embryos. *Gpr161^f/f^* (hereafter *flox* control) mice did express low frequency (∼4.55%) of phenotypic malformations (Fig. 1B, Table 1). We observed kinked tails in *Gpr161^f/+^;Pax3-Cre/+* (hereafter *Cre* control) mice at a very low frequency (Fig. S1), suggesting the existence of a genetic interaction between *Gpr161* and *Pax3* during mouse tail elongation. In contrast, *Gpr161^f/f^;Pax3-Cre* (hereafter *Gpr161* cKO) fetuses showed both craniofacial defects and spinal neural tube malformations (Fig. 1A) with up to 93% penetrance (Fig. 1B, Table 1). With respect to craniofacial defects, we observed tectal hypertrophy, enlarged forebrain and facial defects, initially observed at E13.5, including microphthalmia/anophthalmia and microtia, some of which were phenocopied in *Gpr161* cKO mice with *Wnt1-Cre* in our previous study (Kim et al., 2021). In addition, we observed the spinal dilation starting at E13.5 and various degrees of SB at E17.5 and E18.5 in *Gpr161* cKO fetuses (Fig. 1A, Table 1). There were two representative phenotypic malformations in the spinal regions of *Gpr161* cKO fetuses at E17.5 or E18.5: some fetuses showed skin covered without a protrusive sac at the lumbar regions (4/5), whereas one affected fetus showed a skin-covered lesion with a protrusive sac at the same region (1/5) (Fig. 1A). Of note, we could not observe any live-born *Gpr161* cKO pups, suggesting that *Gpr161* cKO fetuses are embryonic lethal right before birth or possibly during the birth.

**Table 1.**
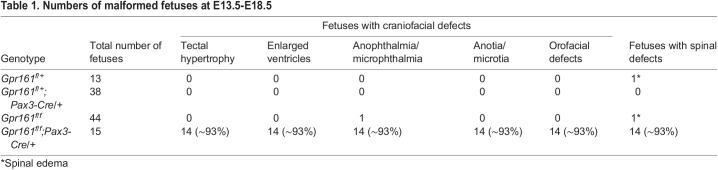
Numbers of malformed fetuses at E13.5-E18.5

**Fig. 2.**
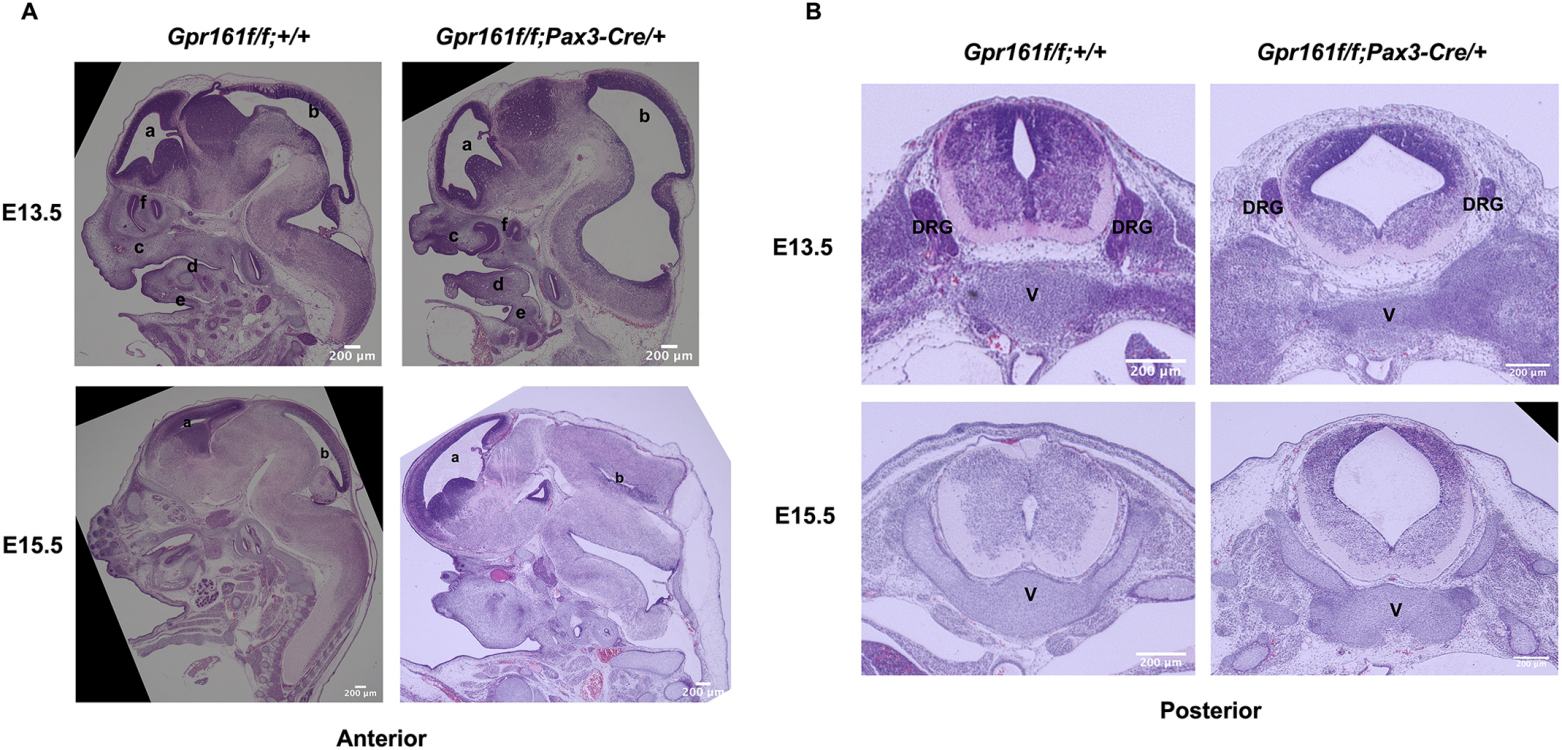
**Histological analysis of *Gpr161^f/f^* and *Gpr161^f/f^;Pax3-Cre/+* fetuses at E13.5 and E15.5.** (A) H&E staining of the craniofacies of *Gpr161^f/f^* (*flox* control) and *Gpr161^f/f^;Pax3-Cre/+* (*Gpr161* cKO) fetuses at E13.5 (*n*=2) (top) and E15.5 (*n*=2) (bottom). ‘a’ and ‘b’, ventricles, ‘c’, maxilla; ‘d’, tongue; ‘e’, mandible; f, nasal cavity. (B) H&E staining with the posterior portion of *flox* control and *Gpr161* cKO fetuses at E13.5 (*n*=2) (top) and E15.5 (*n*=2) (bottom). DRG, dorsal root ganglia; V, vertebrae primordium. Scale bars: 200 µm.

The histological analysis of anterior and posterior portions of the fetuses was performed to examine the gross structural phenotypic malformations of *Gpr161* cKO fetuses (Fig. 2). The tectum and lateral ventricles of *Gpr161* cKO fetuses were extended, and the maxilla and mandibles were hypoplastic (Fig. 2A). These facial malformations were phenocopied in *Gpr161* cKO mice with *Wnt1-Cre* (Kim et al., 2021). At the lumbar level of the spinal cord, the thickness of the neural tubes of *Gpr161* cKO fetuses was reduced compared to that of control littermates, and cystic dilation of the lumens of the spinal neural tube was observed in *Gpr161* cKO fetuses at both E13.5 and E15.5 (Fig. 2B). The dorsal root ganglia were smaller and abnormally positioned in *Gpr161* cKO fetuses and the meninges were observed above the dorsal neural tubes of *Gpr161* cKO fetuses at E13.5 (Fig. 2B). At E15.5, the epidermis covering the spinal neural tubes was thinner. The primordium of vertebral body was reduced and misshaped, and the primordia of vertebral arches and their dorsal ends bent laterally in *Gpr161* cKO fetuses (Fig. 2B), suggesting that the vertebral arches potentially failed to close dorsally. Both the meninges and the disoriented vertebral arches point to the histological characteristics of SB in *Gpr161* cKO fetuses observed in Fig. 1.

### Gpr161 regulates craniofacial and vertebral bone development

**Fig. 3.**
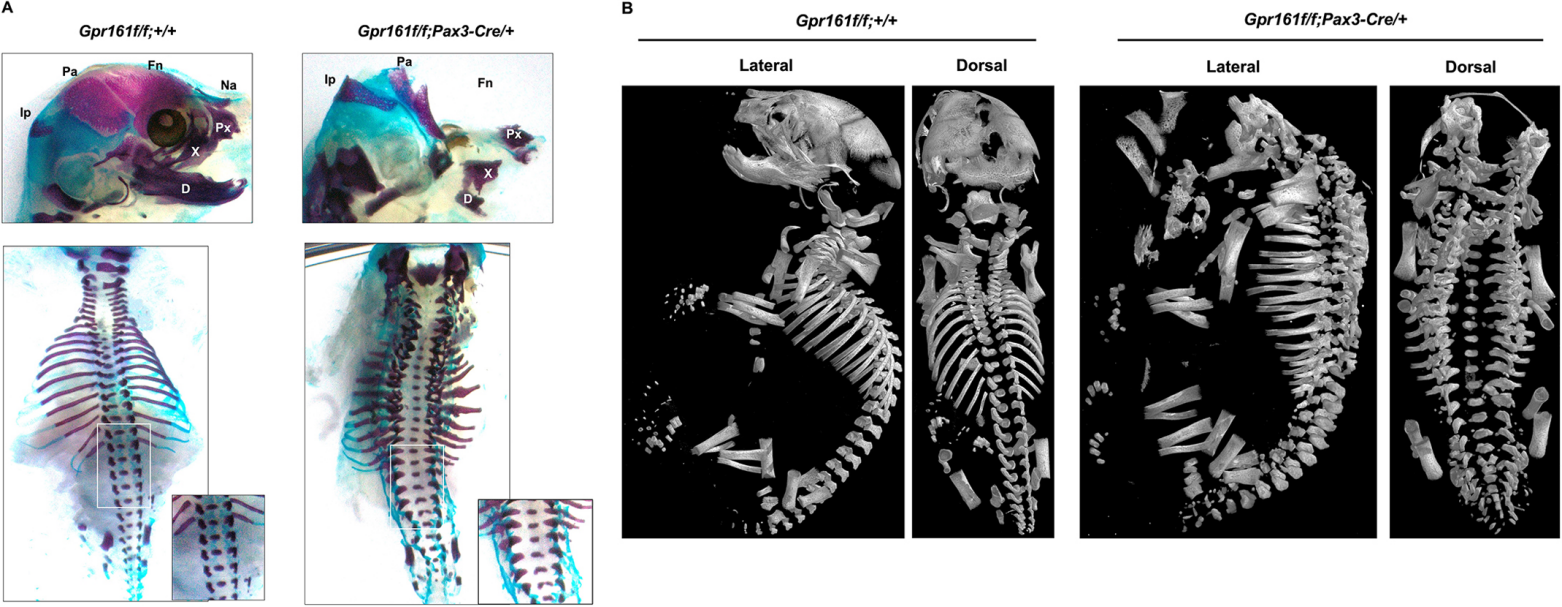
**Skeleton analysis in *Gpr161* cKO fetuses at E17.5.** (A) Visualization of the skeleton of *Gpr161^f/f^* (*flox* control) (*n*=3) and *Gpr161^f/f^;Pax3-Cre/+* (*Gpr161* cKO) (*n*=3) fetuses at E17.5 with Alcian Blue and Alizarin Red staining. Upper panel: craniofacial skeletal staining. D, mandible; Fn, frontal bone; Ip, Interparietal bone; Na, nasal bone; Pa, parietal bone; Px, premaxilla; X, maxilla. Lower panel: axial skeleton staining. (B) Three-dimensional reconstruction of whole-body skeletons of *flox* control (*n*=3) and *Gpr161* cKO (*n*=3) fetuses at E17.5 with microcomputed tomography scans. Lateral and dorsal views of fetuses with each genotype are shown. Scale bars were not available for the images shown.

Shh signaling is involved in the development of the craniofacial bones by regulating cranial neural crest lineages (Jeong et al., 2004), whereas *Gpr161* deletion in cranial neural crest lineages resulted in severe defects in the cranial vault and facial bone formation (Kim et al., 2021). Cre is expressed in the cranial neural crest lineages as well in *Pax3-Cre* (Engleka et al., 2005) and the gross phenotypic malformations in anterior regions of *Gpr161* cKO fetuses were phenocopied in *Gpr161* cKO fetuses with *Wnt1-Cre*. Therefore, to observe the absence or reduction of mineralized cartilage and bones derived from cranial neural crest lineages (frontal, maxilla and mandibles) (Fig. 3A), we performed Alcian blue (unmineralized cartilage)-Alizarin Red (mineralized cartilage and bones) double staining. Much like *Gpr161* cKO embryos with *Wnt1-Cre*, the region of the bone-derived mesodermal lineages (parietal bone) was also reduced. More intriguingly, spinal columns and ribs were substantially misaligned and disorganized along the entire vertebral column from the cervical to sacral regions, and the neural canal was widened in *Gpr161* cKO fetuses at E17.5 (Fig. 3). The vertebral arches were stretched laterally and the cartilage regions between vertebrae were fused in *Gpr161* cKO fetuses, whereas *flox* control fetuses had well-separated vertebrae (Fig. 3A). To confirm the alterations observed by our skeleton-cartilage double staining method, we performed microcomputed tomography to determine the status of skeletal development and observed the same skeletal malformations, including the hypoplasia of craniofacial bones and disorganized vertebrae formation (Fig. 3B), demonstrating a critical role of Gpr161 in somite-derived vertebral development as well as in CNCC-derived cranial vault and facial bone development.

### The molecular basis of phenotypic malformations in *Gpr161* cKO mice

**Fig. 4.**
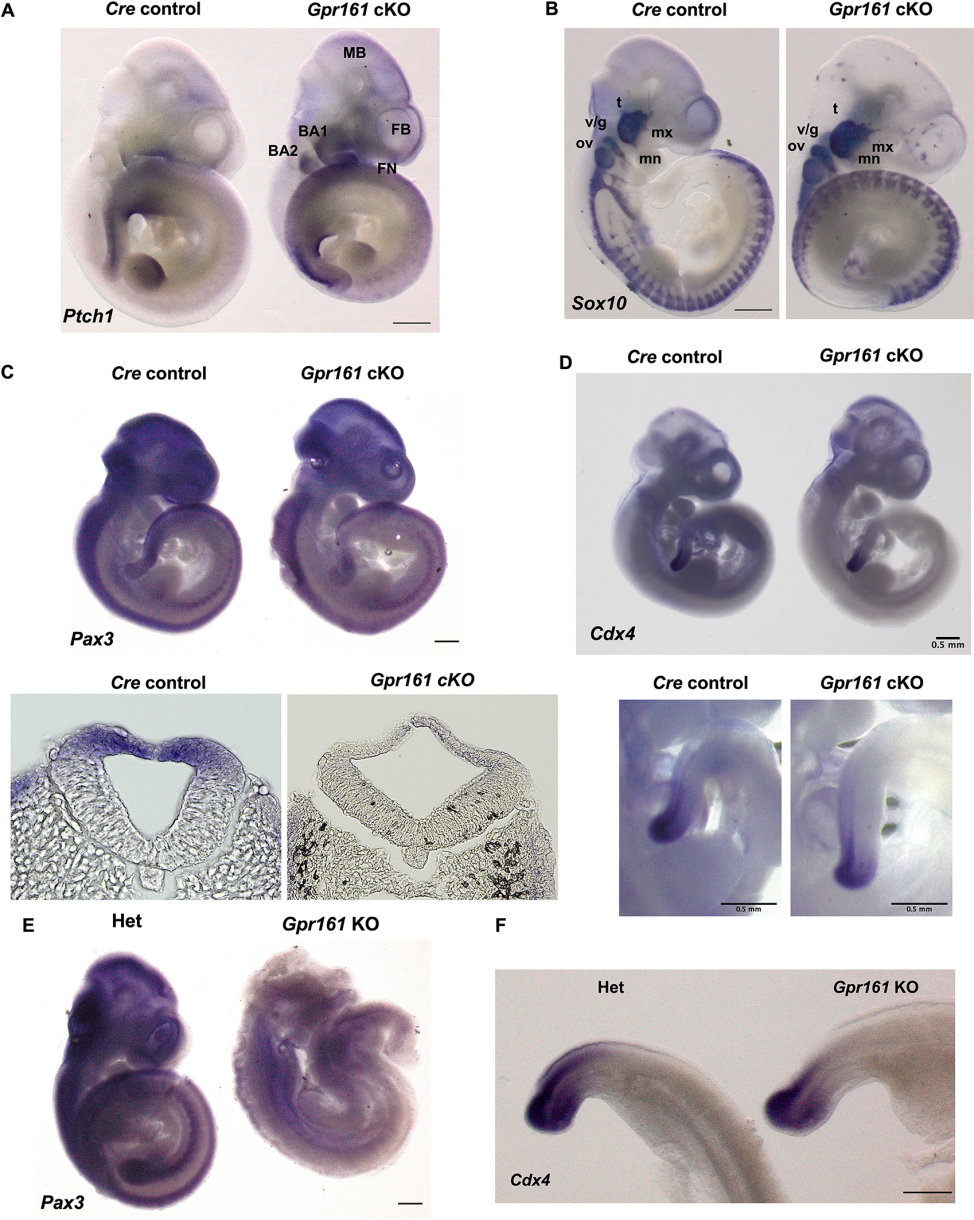
**Expression of dorsal/caudal neural tube markers in *Gpr161* mutant embryos.** (A-D) *Gpr161^f/+^;Pax3-Cre/+* (*Cre* control) and *Gpr161^f/f^;Pax3-Cre/+* (*Gpr161* cKO) embryos at E10.5 were used for whole-mount *in situ* hybridization with antisense probes against (A) *Ptch1* (*n*=3), (B) *Sox10* (*n*=3), (C) *Pax3* (*n*=3) and (D) *Cdx4* (*n*=3). In A: BA1, branchial arch 1; BA2, branchial arch 2; FB, forebrain; FN, frontonasal prominence; MB, midbrain. In B: mn, mandibular branch; mx, maxillary branch; ov, optic vesicle; t, trigeminal ganglion; v/g, vestibulo-cochlear/geniculate ganglia. Transverse sections from embryos with *Pax3* staining are shown in the lower panel in C. The magnified images of tail buds of embryos are shown in the lower panel in D. (E,F) *Gpr161^+/−^* (Het) and *Gpr161^−/−^* (KO) embryos at E10.5 were used for whole-mount *in situ* hybridization with antisense probes against (E) *Pax3* (*n*=3) and (F) *Cdx4* (*n*=3). All scale bars: 0.5 mm. Scale bars were not available for the lower panels in C.

In our previous studies (Kim et al., 2019, 2021), we speculated upon the molecular basis of craniofacial malformations in *Gpr161* cKO fetuses with *Wnt1-Cre*, which phenocopied the craniofacial phenotypes of *Gpr161* cKO fetuses with *Pax3-Cre*. In the present study, we rigorously pursued the molecular basis of the spinal neural tube malformations in *Gpr161* cKO mice, which is the distinguishing phenotypic malformation observed in *Gpr161* cKO mice with *Pax3-Cre*. First, we determined *Ptch1* expression in *Gpr161* cKO embryos via whole-mount *in situ* hybridization to check Shh signaling activity (Mukhopadhyay et al., 2013). *Ptch1* expression was increased overall except in limb buds in *Gpr161* cKO embryos at E10.5 and it was especially highly expressed in the forebrain, midbrain, frontonasal prominence, first and second branchial arches and the spinal neural tube (Fig. 4A). Specifically, *Ptch1* expression was substantially increased at the distal extreme of the caudal neural tube in *Gpr161* cKO embryos (Fig. 4A). *Sox10*, a post-migratory neural crest marker, was expressed in the somites and the branchial arches in both *Cre* control and *Gpr161* cKO embryos without any substantial expression changes, whereas *Sox10* expression in the forebrain and frontonasal prominence was slightly decreased (Fig. 4B), indicative of the prominent role of Gpr161 in CNCC migration during craniofacial development. Our previous transcriptomic analysis showed that *Pax3* gene expression was decreased in *Gpr161* KO embryos (Kim et al., 2019), and Pax3 has been shown to be expressed in the dorsal neural tube during neurulation (Goulding et al., 1991), leading us to examine the involvement of *Pax3* gene regulation. The overall expression of *Pax3* was substantially decreased in *Gpr161* cKO embryos (Fig. 4C) and specifically in the entire dorsal neural tube, somites, forebrain and the frontonasal prominence, and was negatively correlated with *Ptch1* expression (Fig. 4A,C). We further observed a substantial decrease of *Pax3* gene expression in the dorsal spinal neural tube in sectioned *Gpr161* cKO embryos (Fig. 4C, lower panel). In addition, the expression of *Cdx4*, a caudal neural tube marker, was decreased in the tail bud and dorsal neural tube of *Gpr161* cKO embryos (Fig. 4D), suggesting a role for Gpr161 in spinal neurulation and tail elongation. To further confirm the Gpr161-mediated regulation of *Pax3* gene expression, we performed whole-mount *in situ* hybridization with *Gpr161* KO embryos. As expected, *Pax3* expression was substantially decreased in entire *Gpr161* KO embryos compared to its expression in heterozygotes (Fig. 4E), and specifically in the dorsal side of neural tubes and somites, confirming the regulation of *Pax3* gene expression by Gpr161. Similarly, the expression of *Cdx4* was diminished in the tail bud and dorsal spinal neural tube of *Gpr161* KO embryos (Fig. 4F).

### Wnt/β-catenin signaling is involved in regulating *Pax3* gene expression in *Gpr161* cKO embryos

**Fig. 5.**
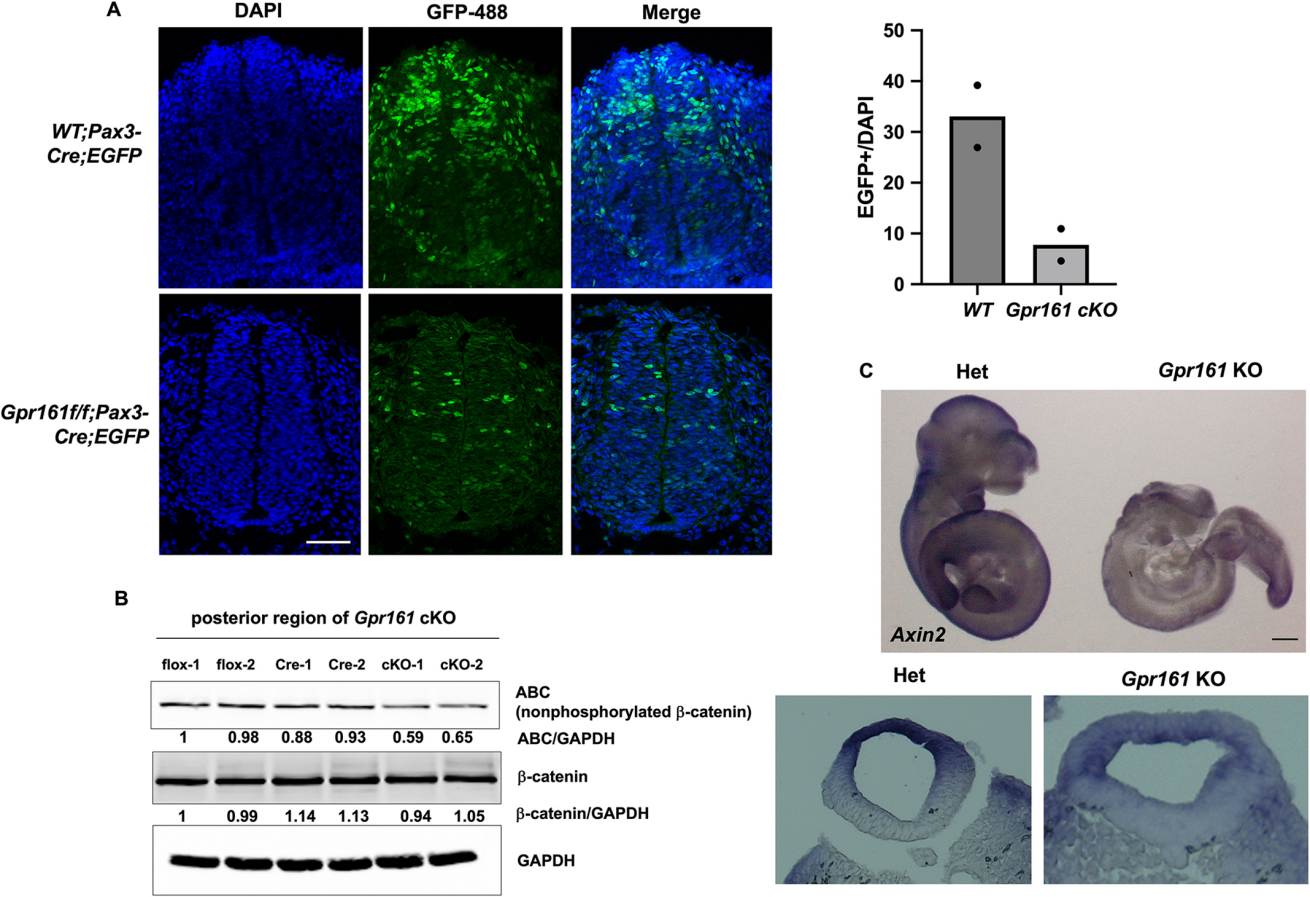
**Wnt signaling activity in the caudal neural tubes in *Gpr161* mutant embryos.** (A) Immunostaining of cryo-sectioned *Cre* control (wild-type or WT) (*n*=2) and *Gpr161* cKO (*n*=2) embryos harboring the *Tcf/Lef1;H2BB-EGFP* allele from different litters. The anti-GFP antibody conjugated with Alexa Fluor 488 was used for EGFP staining and DAPI for staining of the nucleus. Right: quantification of the percentage of the number of EGFP-positive cells relative to the number of DAPI-positive cells in the neuroepithelium. The dots represent the quantifications from two biological replicates. (B) Lysates were prepared from the posterior part of *flox* control, *Cre* control and *Gpr161* cKO embryos at E10.5 from different litters, and then western blotting was performed with antibodies against active β-catenin (ABC), β-catenin and GAPDH. The numbers below the ABC and β-catenin blots indicate the normalized amounts of ABC and β-catenin against GAPDH. Blots represent two biological repeats. (C) E10.5 *Gpr161^+/−^* (Het) and *Gpr161^−/−^* (KO) embryos were used for whole-mount *in situ* hybridization with *Axin2* probes (*n*=4). The transverse sections from embryos with *Axin2* staining are shown in the lower panel. Scale bars: 50 µm (A); 0.5 mm (C, top). Scale bars were not available for the lower panels in C.

We were interested in understanding how *Gpr161* depletion decreased *Pax3* gene expression in the developing mouse spinal neural tube. Previous reports demonstrated that Wnt/β-catenin signaling regulates *Pax3* expression via transcriptional regulation during mouse caudal neural tube closure (Zhao et al., 2014). Our previous study supported the involvement of Gpr161 in the regulation of Wnt/β-catenin signaling during mouse embryonic development (Kim et al., 2019). Therefore, we hypothesized that Gpr161 regulates *Pax3* gene expression via Wnt/β-catenin signaling during caudal neural tube development. To evaluate Wnt/β-catenin signaling activity in *Gpr161* cKO embryos, we first used Wnt reporter mice (*Tcf/Lef1;H2BB-EGFP*; Ferrer-Vaquer et al., 2010) with *Gpr161* cKO. The EGFP signal in the dorsal neural tube of *Gpr161* cKO embryos harboring *Tcf/Lef1;H2BB-EGFP* was decreased (Fig. 5A), suggesting that Wnt/β-catenin signaling activity was reduced in the dorsal neural tube of *Gpr161* cKO embryos. In addition, we observed decreased levels of active β-catenin (ABC; non-phosphorylated β-catenin), another indicator of canonical Wnt signaling activity, in the posterior region of *Gpr161* cKO embryos, whereas the total β-catenin level had not changed (Fig. 5B). To further confirm Gpr161-mediated regulation of canonical Wnt signaling, we analyzed the expression of *Axin2*, a target gene of canonical Wnt signaling, in *Gpr161* KO embryos (Fig. 5C). Overall *Axin2* expression was diminished in *Gpr161* KO embryos, particularly in the dorsal aspect of the neural tube (Fig. 5B; lower panel), demonstrating that canonical Wnt signaling activity was decreased during spinal neural tube development in *Gpr161* KO embryos. Our results consistently support the notion that Wnt signaling activity was decreased in the dorsal aspect of the posterior neural tube in *Gpr161* cKO and *Gpr161* KO embryos.

## DISCUSSION

This study revealed the critical role of *Gpr161* in *Pax3* lineages during mouse embryonic development. *Gpr161* cKO mice presented with two distinct patterns of phenotypic malformations. One involved craniofacial defects that included tectal hypertrophy and skeletal anomalies, which was phenocopied in *Gpr161* cKO mice with *Wnt1-Cre* in our previous study (Kim et al., 2021). The second type of altered development involved spinal malformations, including spinal neural tube and vertebral skeletal defects, which represents a novel finding of this study. We subsequently observed increased Shh signaling, decreased expression of caudal/dorsal neural tube markers, *Pax3* and *Cdx4* in *Gpr161* cKO embryos, as visualized by whole-mount *in situ* hybridization. We further observed decreased Wnt/β-catenin signaling in the dorsal side of the spinal neural tube in *Gpr161* cKO and KO embryos with Wnt reporter mice by examining ABC protein levels and *Axin2* gene expression, respectively, indicating the involvement of Wnt signaling in *Pax3* gene regulation (summarized in Fig. S3).

### Craniofacial malformations in *Gpr161* cKO mice

In *Pax3-Cre* mice, Cre is expressed in the dorsal neural tube along the entire anterior-posterior axis and the facial structures at E9 in mouse embryos (Engleka et al., 2005), which is similar to the Cre expression pattern found in *Wnt1-Cre* mice with respect to the facial structures. Indeed, protrusive tectal defects and craniofacial skeletal defects were phenocopied in *Gpr161* cKO embryos with *Wnt1-Cre*. Furthermore, we observed increased Ki67 (encoded by *Mki67*) staining in the mesencephalic progenitors in *Gpr161* cKO fetuses at E13.5 (Fig. S2A) and increased *Ptch1* expression in midbrain regions in younger embryos (Fig. 4A), confirming the important role of Shh signaling in midbrain morphogenesis. This was consistent with our previous study with *Gpr161* cKO with *Wnt1-Cre* and *Nestin-Cre* (Kim et al., 2021), and another study with *Ptch1* cKO with *Nestin-Cre* (Martinez et al., 2013). We further identified that *Pax3* expression was decreased in the dorsal mesencephalon in *Gpr161* cKO as well as *Gpr161* KO embryos at E10.5 (Fig. 4C, upper panels; Fig. 4E), providing evidence of the potential regulatory mechanisms of *Pax3* gene expression by Shh signaling during midbrain morphogenesis. This possibility is supported by a previous report (Cairns et al., 2008) that showed that increased Shh signaling inhibited *Pax3* gene expression with coordination of Wnt/β-catenin signaling during somitogenesis in chicken embryos. In addition to tectal hypertrophy, extended lateral ventricles in the forebrain of *Gpr161* cKO fetuses at E15.5 were observed (Figs 1A and 2A), and a similar phenotypic malformation, ventriculomegaly, was described in *Gpr161* cKO mice with *Nestin-Cre* (Shimada et al., 2019). We observed increased Shh signaling and reduced expression of the CNCC markers *Sox10* and *Pax3* in the embryonic forebrain at E10.5, strongly indicating that Gpr161 regulates forebrain morphogenesis via CNCC lineages.

The calvaria and facial bone-derived CNCCs were absent or underdeveloped, and bones derived from paraxial mesodermal cells were also underdeveloped, but to a lesser degree (Fig. 3). These results confirm the role of Gpr161 in the intramembranous ossification during CNCC-derived cranial vault formation and facial skeletogenesis. It is consistent with our and others’ previous observations (Hwang et al., 2018; Kim et al., 2021; Li et al., 2017) that increased Shh signaling in CNCCs and mesoderm lineages inhibits calvaria and facial bone formation by dysregulating mesenchymal condensation in the frontonasal and facial prominences. The inhibition of calvaria and facial bone formation was more prominent in *Gpr161* cKO mice compared to that in *Sufu* cKO mice with *Wnt1-Cre*, suggesting the involvement of other signaling pathways during Gpr161-mediated craniofacial skeletogenesis. Indeed, we observed not only increased *Ptch1* expression in the frontonasal prominence and branchial arches of *Gpr161* cKO embryos, but also decreased *Axin1* expression in the same areas of *Gpr161* KO embryos, suggesting the involvement of Wnt/β-catenin signaling in Gpr161-mediated craniofacial skeletal development.

### Spinal malformations in *Gpr161* cKO mice

The most distinct phenotypic malformation observed in the *Gpr161* cKO embryos compared to *Gpr161* cKO embryos with *Wnt1-Cre* was the spinal neural tube malformation with vertebral defects. Although Wnt1 and Pax3 are well known neural crest cell markers, Cre expression patterns between *Wnt1-Cre* and *Pax3-Cre* are slightly different; Cre is expressed in the craniofacial regions at E9.5 in *Wnt1-Cre* from our previous study (Kim et al., 2021), whereas Cre is expressed more widely from the anterior to the posterior neural tubes in *Pax3-Cre* at the same embryonic stage (Zhao et al., 2014). This may result in the differential phenotypic malformations, i.e. spinal neural tube malformation, in *Gpr161* cKO embryos compared to *Gpr161* cKO embryos with *Wnt1-Cre*. The spinal neural tube malformation in *Gpr161* cKO embryos was not a neural tube closure defect, as we could not observe any obvious posterior neuropore (PNP) opening (only less than 5% embryos) right after neurulation (at E10.5) and the histological analysis of the spinal neural tube of *Gpr161* cKO fetuses (Fig. 2B) did not reveal any indication of an open neural tube, suggesting that it could be the closed form of SB. There were discrepant spinal phenotypic malformations between *Gpr161* cKO and *Gpr161* KO embryos: *Gpr161* cKO showed very few open PNPs, whereas *Gpr161* KO had open PNPs with full penetrance at E10.5. One possible explanation could be the timing and the degree of *Gpr161* depletion, which potentially affects critically relevant gene expression during spinal neural tube closure. This possibility is supported by *Pax3* gene expression patterns: whereas *Pax3* expression was decreased in entire embryos, there was a more prominent reduction in expression in the spinal neural tube in *Gpr161* KO compared to that in *Gpr161* cKO (Fig. 4C,E), unlike another caudal marker, *Cdx4*, which was similarly decreased in embryos of both genotypes. These results further suggest that Gpr161 is associated with both open and closed SB by regulating the expression of critical genes in murine spinal neurulation, such as *Pax3*.

A previous report revealed that the transcriptional regulation of *Pax3* by Wnt/β-catenin signaling is critical for β-catenin-mediated posterior neural tube closure (Zhao et al., 2014), which led us to examine the involvement of Wnt/β-catenin signaling in Gpr161-mediated *Pax3* gene regulation. Our observation in Fig. 5 of reduced Wnt/β-catenin signaling activity in the dorsal spinal neural tube in *Gpr161* cKO and KO embryos suggests that Wnt/β-catenin signaling contributes to a potential regulatory mechanism for Gpr161-mediated *Pax3* gene regulation. In addition, Cairns et al. (2008) demonstrated that the *Pax3* gene is induced by the Wnt morphogen secreted from the dorsal neural tube and surface ectoderm, and it was repressed with increased Shh gradient by inducing Nkx3.2 (encoded by *Nkx3-*2) during somitogenesis, providing an alternative regulatory mechanism of Gpr161-mediated *Pax3* gene regulation via Shh signaling.

Unlike elevated proliferation in the mesencephalon of *Gpr161* cKO mice, we failed to observe any proliferation changes in the spinal neuroepithelium of *Gpr161* cKO fetuses (Fig. S2B), suggesting the involvement of other cellular defects during the spinal neurulation in *Gpr161* cKO mice. Increased apoptosis was observed in the neural tube of *Splotch* mice (Phelan et al., 1997), which was rescued by inhibition of p53-mediated apoptosis (Pani et al., 2002). In addition, Ptch1 is reported to have a pro-apoptotic function in the developing spinal neural tube in mice (Thibert et al., 2003), suggesting the potential role of apoptosis in Gpr161-Pax3-mediated spinal neural tube formation.

The skeletal defects of paraxial mesoderm derived-vertebral columns and ribs in *Gpr161* cKO fetuses (Fig. 3) further suggest the role of Gpr161 in the mesoderm-derived endochondral skeletogenesis, which is supported by a previous study with *Prx1-Cre* as the driver (Hwang et al., 2018). The question remains as to whether the vertebral column defects observed in *Gpr161* cKO fetuses are some of the underlying causes of the closed forms of SB phenotypes.

### Summary and implication to human diseases

This study revealed the important role of Gpr161 in craniofacial morphogenesis and skeletogenesis, as well as in spinal neural tube morphogenesis and vertebral formation during mouse embryonic development. We further provide the molecular pathogenetic function of Gpr161 in the regulation of *Pax3* gene expression involving Wnt/β-catenin signaling during spinal neural tube formation. Our study provides a novel closed SB mouse model in the field and brings a new insight regarding its molecular pathogenesis via *Pax3* gene regulation involving distinct regulation of Shh and Wnt signaling during spinal neurulation. Based on the implication of *Gpr161* in both open (KO) and closed (cKO) SB, our study provides a research model to study the differential molecular profiles between open and closed SB and the potential genetic implication of *GPR161* in human closed SB, which has been rarely studied, beyond its implication in open SB that we previously reported (Kim et al., 2019). Lastly, our results suggest a potential molecular target to develop intervention strategies for the prevention of the closed form of SB with specific genetic mutations. One of these targets can be the genetic or chemical modulation of Wnt/β-catenin signaling and/or the *Pax3* gene to rescue the abnormal phenotypes in *Gpr161* mutant mice, which will be the focus of our future studies.

## MATERIALS AND METHODS

### Mouse strains

All mice were maintained according to the guidelines approved by the Institutional Animal Care and Use Committee (IACUC) of The University of Texas at Austin. *Gpr161* cKO (*Gpr161^f/f^* or *Gpr161 flox*) and KO (*Gpr161^−/−^*) mice were graciously provided by Dr Saikat Mukhopadhyay [University of Texas Southwestern Medical Center (UT Southwestern), Dallas, TX, USA] and detailed information concerning the generation of transgenic lines has been previously reported (Hwang et al., 2018). The transgenic mice *Pax3-Cre* (#005549) and *Tcf/Lef1;H2BB-EGFP* (#013752) were purchased from Jackson Laboratory. The genotypes of the mice and embryos/fetuses were determined by PCR-based genotyping.

### Tissue processing and immunostaining

Embryos were collected at E10.5 from timed matings and processed for cryo-sectioning. To this end, the embryos from different litters were fixed, incubated in 30% sucrose solution at 4°C until they were submerged, and mounted into optimal cutting temperature (OCT) solution. OCT-embedded embryos were cryo-sectioned using a Cryotome (Thermo Fisher Scientific) with 10 µm thickness for immunostaining. The frozen sections were incubated with blocking buffer (10% goat serum in PBS) followed by washing with PBS. Subsequently, they were incubated with Alexa Fluor 488-conjugated anti-GFP antibody (1:200, A-21311, Thermo Fisher Scientific) in blocking buffer and then with DAPI (1 µg/ml) prior to mounting. Images were captured using a Nikon Ti2E/CSU-W1 spinning-disc confocal microscope. For whole-mount *in situ* hybridization, paraffin-embedded embryos were sectioned at 10 µm thickness, deparaffinized, and the slides were visualized using an Olympus SZX2-ILLT microscope (Olympus, Tokyo, Japan). The EGFP- and DAPI-positive cells in the neuroepithelium of neural tubes were counted with ImageJ/Fiji software (National Institutes of Health).

### Immunohistochemistry

Fetuses were harvested at E13.5 from timed matings between *Gpr161^f/f^* and *Gpr161^f/+^;Pax3-Cre/+.* Collected fetuses were fixed, paraffin embedded and sectioned with 4 μm thickness. The paraffin-embedded sections were deparaffinized, dehydrated, antigen-retrieved, blocked (blocking solution, Thermo Fisher Scientific), and incubated with primary antibody [anti-Ki67, 9027, Cell Signaling Technology; diluted 1:200 with Lab Vision Antibody Diluent Quanto (Thermo Fisher Scientific)] overnight at 4°C. After washing, sections were incubated with horseradish peroxidase (HRP) polymer conjugate (UltraVision LP detection system, Thermo Fisher Scientific) and 3,3′-diaminobenzidine (DAB; BosterBio). The sections were counterstained with Hematoxylin (Thermo Fisher Scientific). Images were captured using an All-In-One Fluorescence microscope (Keyence) using a 2× and 20× objective. The images were analyzed with ImageJ/Fiji software.

### Whole-mount *in situ* hybridization

Embryos were collected at E10.5 from timed matings either between *Gpr161^f/f^* and *Gpr161^f/+^;Pax3-Cre/+* or between *Gpr161* heterozygotes (*Gpr161^+/−^*). The collected embryos were fixed and dehydrated with methanol and were then pooled based on their genotypes for further analysis. Whole-mount *in situ* hybridization was performed according to standard protocols (Wei et al., 2011). The cDNA plasmids targeting *Pax3* and *Axin2* were obtained from Lee Niswander (University of Colorado), *Sox10* from Yoshihiro Komatsu (McGovern Medical School, The University of Texas Health Science Center at Houston), and *Ptch1* from Steven Vokes (The University of Texas at Austin). The DNA template for RNA probes targeting *Cdx4* were generated (Piette et al., 2008) based on the sequence information from the Allen Institute (primers for DNA templates of RNA probes: *Cdx4*, forward primer, 5′-AGTTTACAGGGACCTCAGGATG-3′; reverse primer, 5′-CAAGAGAAACCAGTGACTCG-3′). Images were captured with the Olympus SZX2-ILLT microscope.

### Western blotting

Embryos were harvested at E10.5 from timed matings between *Gpr161^f/f^* and *Gpr161^f/+^;Pax3-Cre/+* mice. The posterior part of embryos from different litters was collected by cutting at the level of hindlimb buds and was lysed with radioimmunoprecipitation assay (RIPA) buffer (BP-115, Boston BioProducts). The lysates were used for western blotting with anti-ABC (1:1000, 8814, Cell Signaling Technology), anti-β-catenin (1:2000, 610163, BD Biosciences) and anti-GAPDH (1:5000, 2118, Cell Signaling Technology) antibodies, and then with 1RDye 800CW goat anti-rabbit IgG (LiCOR). The images were captured using an Odyssey imaging system (LI-COR). The band intensity was quantified with ImageJ software.

### Bone-cartilage skeletal staining

Skeletal staining was performed using a modified Alcian Blue/Alizarin Red staining procedure (Kessel et al., 1990). Briefly, E17.5 fetuses collected from different litters were eviscerated and fixed with 95% ethanol and then with acetone. Fixed fetuses were incubated with staining solution (0.005% Alizarin Red S, 0.015% Alcian Blue GS in 5% acetic acid, 5% H_2_O and 90% ethanol) for 3 days at 37°C. After washing, samples were kept in 1% KOH for 48 h. For long-term storage, specimens were serially transferred into 20%, 50% and 80% glycerol solutions and were ultimately maintained in 100% glycerol. The images were captured with the Olympus SZX2-ILLT microscope.

### Microcomputed tomography and image processing

E17.5 fetuses collected from different litters were fixed with 10% formalin, followed by fixation with 70% ethanol. Specimens were scanned at the University of Texas High-Resolution X-ray Computed Tomography Facility using the flat panel detector on a Zeiss Xradia 620 Versa microscope. The X-ray source was set to 70 kV and 8.5 W with no filter. A total of 2001 0.1 s projections were acquired over ±180° of rotation with no frame averaging. A source-object distance of 18.0 mm and a detector-object distance of 251.7 mm resulted in 9.98 μm resolution.

## Supplementary Material

10.1242/dmm.050277_sup1 Supplementary information
