# Supplementary material for: Pax3 lineage-specific deletion of Gpr161 is associated with spinal neural tube and craniofacial malformations during embryonic development
Source: Dis Model Mech. 2023 Nov 28;16(11):dmm050277. doi: 10.1242/dmm.050277 (PMC10694864; doi:10.1242/dmm.050277)
Supplement: Supplementary information [file dmm-16-050277-s1.pdf]

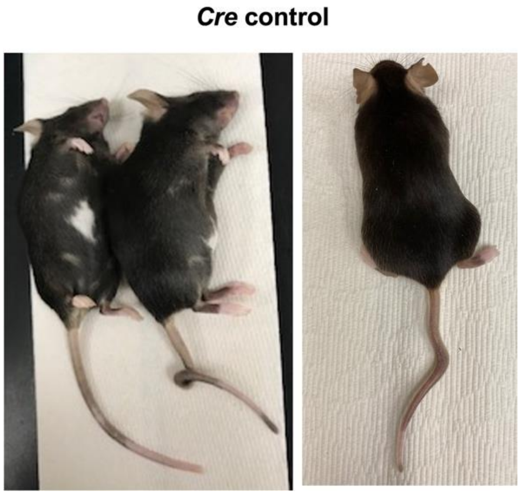

Fig. S1. The kinked/curly tail in *Cre* control (*Gpr161*$^{f/+}$*;Pax3-Cre/+*) adult mice.

Disease Models & Mechanisms • Supplementary information

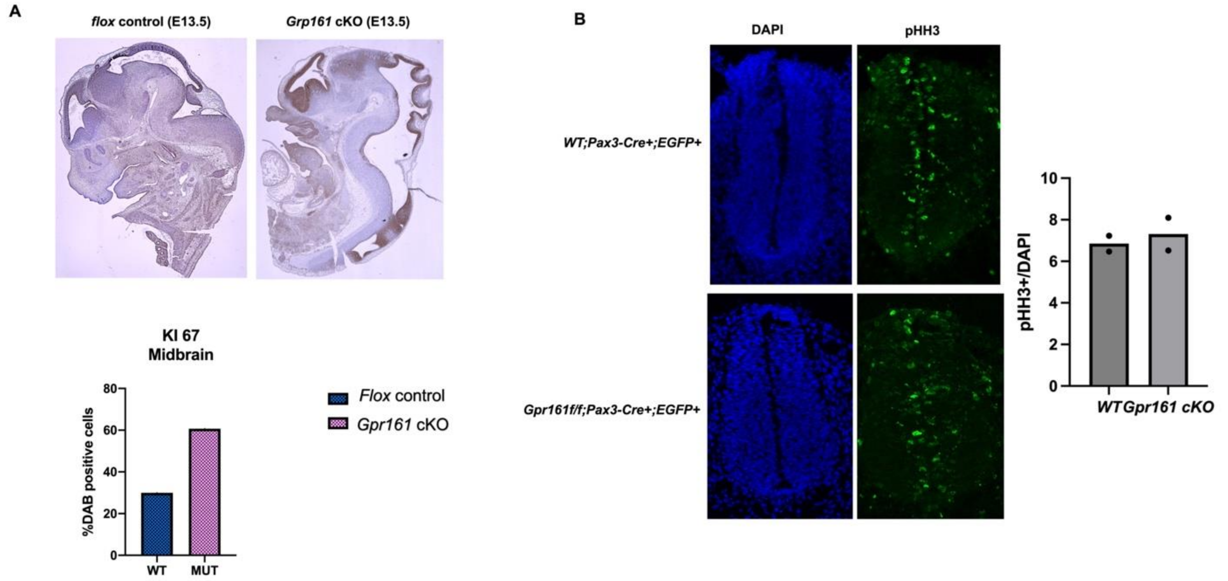

Fig. S2. The status of cell proliferation of dorsal midbrain and spinal neural tubes in *Gpr161* cKO. (A) Paraffin-embedded sections from anterior part of fetuses of *flox* control (n=2) and *Gpr161* cKO (n=2) at E13.5 were used for IHC. Upper panel: Ki67 staining, Lower panel: Analysis of Ki67 positive cells in dorsal midbrain. (B) Cryo-sections from *WT;Pax3-Cre;Tcf4/Lef1:H2BB-EGFP/+* (n=2) and *Gpr161*$^{f/f}$*;Pax3-Cre;Tcf4/Lef1:H2BB-EGFP/+* (n=2) were used for immunostaining with phospho Histone H3 (pHH3) and β-catenin. The nucleus was stained with 4′,6-diamidino-2-phenylindole (DAPI). Right: The quantification bars represent the percentage of pHH3 positive cells out of DAPI positive cells in neuroepithelium. Each dot represents the quantification from two biological replicates.

Disease Models & Mechanisms • Supplementary information

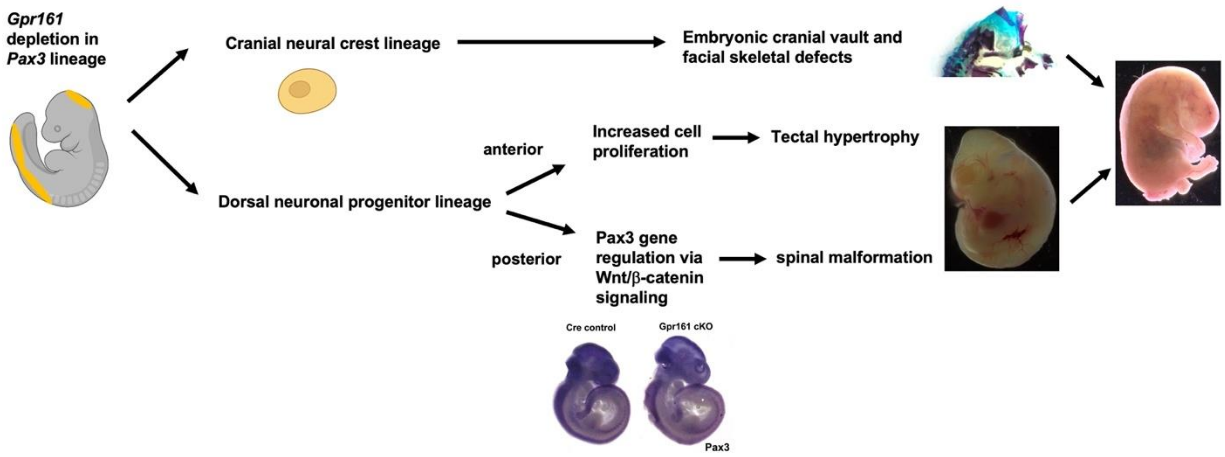

Fig. S3. Schematic Summary. The *Pax3*-lineage specific Cre is expressed in the cranial neural crest cells and dorsal neural progenitor cells. *Gpr161* cKO with *Pax3-Cre* has two distinct phenotypes; one is craniofacial defects that is phenocopied with *Gpr161* cKO with *Wnt1-Cre* and the other is closed spina bifida, which is the novel phenotype in *Gpr161* cKO with *Pax3-Cre*. The spinal neural tube malformation results from the reduced *Pax3* gene expression involving reduced Wnt/β-catenin signaling activities in *Gpr161* cKO with *Pax3-Cre*. The anterior phenotypic malformation results from the increased cell proliferation and cranial vault and facial bone defects as we observed in *Gpr161* cKO with *Wnt1-Cre*. We utilized BioRender to draw this scheme.

Disease Models & Mechanisms • Supplementary information
